# *Plasmodium vivax* in Papua New Guinea: high diversity and gene flow among endemic populations signal roadblocks for elimination

**DOI:** 10.1186/1475-2875-11-S1-O24

**Published:** 2012-10-15

**Authors:** Cristian Koepfli, Ivo Mueller, Peter Siba, Ingrid Felger

**Affiliations:** 1 Swiss Tropical and Public Health Institute, Basel, Switzerland; 2 PNG Institute of Medical Research, Goroka, Papua New Guinea; 3 Walter and Eliza Hall Institute, Parkville, Victoria, Australia

## 

The highest prevalence of *Plasmodium vivax* is observed in the lowlands of Papua New Guinea (PNG), the only country in the Western Pacific Region that experienced an increase in malaria cases over the last decade. Contrastingly, prevalence is lower in the Solomon Islands, a country aiming to eliminate malaria, where the number of confirmed cases decreased by approximately 50% from 2000 to 2010.

Population structure can inform interventions against malaria. Genetic diversity, gene flow and linkage disequlibrium (LD) between loci are thought to influence the emergence and spread of drug resistance and may affect efficiency of future vaccines. In endemic areas where transmission has been reduced to low levels, genotyping could play an important role in tracking outbreaks and to identify the origin of imported malaria cases.

In areas of a *P. vivax* endemicity lower than in PNG considerable genetic differentiation between populations was found, suggesting limited gene flow. To understand the population genetic structure of *P. vivax* in the South Pacific, we have used 14 molecular markers to genotype 295 *P. vivax* samples from four sites in PNG and from the Solomon Islands. Diversity was very high, with expected heterozygosity values ranging from 0.62 to 0.98 for the different markers. As a result, the effective population size was also found to be high. Among the four PNG sites, a near absence of population structure was observed (*F*_sτ_ < 0.015). When comparing PNG and the Solomon Islands, population structure was found to be weak (*F*_sτ_ = 0.03 to 0.044).

*P. vivax* populations in the South Pacific are much less structured than populations from areas of lower endemicity in Latin America and Asia. In PNG, the presence of a large *P. vivax* reservoir seem to overcome the geographical barriers to transmission. Intensified, sustained control programs, which are coordinated among endemic countries and also target asymptomatic carriers, seem to be required to eliminate *P. vivax* in the South Pacific.

